# Evaluation of Lower Urinary Tract Dysfunction Impact on Quality of Life in Multiple Sclerosis Patients: Russian Translation and Validation of SF-Qualiveen

**DOI:** 10.1155/2020/4652439

**Published:** 2020-07-25

**Authors:** Ekaterina S. Philippova, Igor V. Bazhenov, Alexander V. Ziryanov, Ekaterina Y. Moskvina

**Affiliations:** ^1^Department of Urology, Ural State Medical University, Yekaterinburg 620028, Russia; ^2^Regional Urological Center, Sverdlovsk Regional Clinical Hospital No.1, Yekaterinburg 620102, Russia; ^3^Department of Neurology and Neurosurgery, Ural State Medical University, Yekaterinburg 620028, Russia

## Abstract

The Short Form Qualiveen (SF-Qualiveen) is an 8-item version of the Qualiveen questionnaire used to evaluate the impact of urinary symptoms on the quality of life in patients with urological dysfunction due to neurological disorders. The questionnaire was never available in the Russian language before. *The study is aimed* at providing the translation, cultural adaptation, and validation of a Russian version of the SF-Qualiveen for the use in patients with multiple sclerosis (MS). *Materials and Methods*. The original English language version of the SF-Qualiveen was translated into Russian according to the cultural and linguistic adaptation algorithm. The participants (50 MS patients with neurogenic bladder and 10 relatively healthy volunteers) filled out the finalized Russian version of the SF-Qualiveen and the Neurogenic Bladder Symptom Score (NBSS) twice, 2 weeks apart. The data obtained was used to determine the internal consistency (Cronbach's alpha), external validity (the Spearman correlation), and test-retest reliability (intraclass correlation coefficient (ICC)) of the questionnaire. *Results*. The mean SF-Qualiveen total score was 2.51 ± 0.79 in patients with a neurogenic bladder and 0.1 ± 0.02 in the control group (*p* < 0.001). Cronbach's alpha exceeded 0.9 indicating an excellent internal consistency of the questionnaire. The retest did not reveal any significant differences between the findings. The test-retest reliability was good for all items and domains (ICC 0.81-0.89). The total score demonstrated the highest ICC (0.89). The external validity was verified by a strong correlation demonstrated between the SF-Qualiveen and NBSS scores. *Conclusions*. The Russian SF-Qualiveen questionnaire is a reliable, valid, and consistent tool for the assessment of a urinary disorder impact on the quality of life in patients with MS.

## 1. Introduction

A neurogenic bladder (NB) can be a manifestation of any disease of or damage to the nervous system. For instance, about 75% of patients with multiple sclerosis (MS) have lower urinary tract dysfunction [1] presenting storage symptoms (50%), voiding symptoms and urinary retention (25%), or their combination (25%). These symptoms have a significant negative impact on the quality of life in patients with MS [2].

The urological status of patients with neurogenic lower urinary tract dysfunction (NDLUT) should be evaluated comprehensively. The evaluation must include recording of complaints and history, a physical examination, blood and urine tests, and ultrasonic, urodynamic, and endoscopic examinations. To facilitate obtaining a more objective and detailed record of complaints of a patient with lower urinary tract dysfunction, urologists traditionally use bladder diaries and various self-administered questionnaires. The European Association of Urology (EAU) recommends a mandatory evaluation of NDLUT patients' quality of life with the use of validated questionnaires [3].

More than 30 questionnaires intended for the evaluation of neurogenic bladder symptoms and their impact on a patient's quality of life have been made available in English. Only some of them were made specifically for patients with NLUTD [4]; among these, the Qualiveen and the Neurogenic Bladder Symptom Score (NBSS) are the best-known. Initially developed by Costa et al. [5] in French, the Qualiveen was intended to evaluate the impact on the quality of life by lower urinary tract dysfunction in patients with spinal cord injury. It contains 30 questions based on interviews with patients, expert evaluation, and prior studies. Then later, the questionnaire was validated for MS patients [6] and translated into English [7], German [8], Italian [9], Portuguese [10], and Spanish [11]. The Short Form Qualiveen (SF-Qualiveen) developed by Bonniaud et al. [12] contains 8 questions in 4 domains—bother with limitations, fears, feelings, and frequency of limitations. Total scores can range from 0 (no symptoms at all) to 4 (maximum symptoms). Currently, it is available in French, English, German [13], and Polish [14].

The 24-item NBSS was developed by Welk et al. [15, 16] as an objective and validated tool for the assessment of bladder symptoms in patients with neurogenic bladder dysfunction resulting from a spinal cord injury, multiple sclerosis, and spina bifida. This instrument is aimed at measuring symptoms, while neither assign relative values to them nor judge their impact on patients' quality of life. The final question of the NBSS makes a direct inquiry about the person's urinary quality of life. The remaining questions address the 3 domains—incontinence, storage and voiding, and consequences. Total scores can range from 0 (no symptoms at all) to 74 (maximum symptoms). Validated translations into Brazilian Portuguese [17], Italian [18], and Russian [19] have been published.

The aim of this study was to translate, culturally adapt, and validate a Russian version of the SF-Qualiveen for the use in patients with MS.

## 2. Materials and Methods

The study design has two stages. The first stage includes the translation and cross-cultural adaptation of the SF-Qualiveen questionnaire into Russian. The second stage is the assessment of the reliability and validity of the Russian version. The study was approved by the Local Ethics Committee of the Sverdlovsk Regional Clinical Hospital.

### 2.1. Stage 1: SF-Qualiveen Translation and Cross-Cultural Adaptation

Following the copyright holder's (Mapi Research Trust) approval, the NBSS questionnaire had been translated into Russian according to the cultural and linguistic adaptation algorithm [20, 21]. A professional translator (native Russian speaker) translated the English version of SF-Qualiveen into Russian. Then, a team of 5 English-speaking urologists reviewed the translation to assess its precision, content reliability, and similarity to the English version. Later, a Russian-speaking native English speaker backtranslated the Russian version into English. The translators involved in the project agreed that the original text and the Russian version are retranslatable and similar. The resulting Russian version was tested in interviews with 10 patients with MS. Following the ensued discussion, the Russian version of the questionnaire was finalized.

### 2.2. Stage 2: SF-Qualiveen Russian Version Reliability and Validity Assessment

The finalized Russian version of the SF-Qualiveen was tested in a total of 60 subjects (43% males and 67% females) aged 26-58 years (37.9 ± 9.16 years) enrolled. The study and control groups included 50 MS patients with a neurogenic bladder and 10 relatively healthy volunteers, respectively. In the study group 71%, 25%, and 4% of subjects had remittent, secondary progressive, and primary progressive MS, respectively; the history of MS was 12.7 ± 8.6 years long, and the lower urinary tract symptoms' duration was 7.4 ± 5.5 years. Neurologists examined the subjects and scored their disability according to the Expanded Disability Status Scale (EDSS). The EDSS scores were 4.41 ± 0.91. The respondents answered the questions of the Russian SF-Qualiveen and the Russian version of NBSS twice, 2 weeks apart.

### 2.3. Statistical Analysis

The internal consistency was tested using Cronbach's coefficient *α*. The values were interpreted as follows: poor: less than 0.5, moderate: 0.5 to 0.75, good: 0.75 to 0.9, and excellent: more than 0.9 [22, 23].

The short-term test-retest reliability of the Russian version of the SF-Qualiveen was assessed using the intraclass correlation coefficient (ICC). The ICC values were interpreted as follows: poor: less than 0.5, moderate: 0.5 to 0.75, good: 0.75 to 0.9, and excellent: more than 0.9 [23, 24].

The construct's validity was measured by examining the relationship between SF-Qualiveen and NBSS by domains and the total scores using Spearman's correlation coefficient. The values were interpreted as follows: excellent: at least 0.9, high: 0.7 to 0.89, moderate: 0.50 to 0.69, fair: 0.26 to 0.49, and little or no relationship: less than 0.25 [24].

The demographic data and the variables of the statistical analyses were expressed as the mean ± standard deviation (SD). The data was analyzed with the use of the SPSS 22.0 software.

## 3. Results

The mean overall SF-Qualiveen score in MS patients with NLUTD (see Table 1) was 2.51 ± 0.79. This significantly (*p* < 0.001) exceeded the scores of controls (0.1 ± 0.02) [22]. The test-retest reliability was good for all items and domains (ICC 0.81-0.89). The total scores demonstrated the highest ICC (0.89).

SD: standard deviation; ICC: intraclass correlation coefficient.

Cronbach's alpha for the total score and the feelings domain was more than 0.9 indicating an excellent internal consistency of the questionnaire (see Table 2). The bother with limitations domain demonstrated good, and the frequency of limitations, a moderate internal consistency. The fears domain showed poor consistency. Retest did not result in any significant differences in the internal consistency.

The questionnaire demonstrated a high external validity (see Table 3). The SF-Qualiveen scores correlated with the NBSS scores with high reliability. A significantly strong correlation was demonstrated between the quality of life item of NBSS, the SF-Qualiveen total score, and the bother with limitations and feelings domains. Most of the SF-Qualiveen domain scores showed a moderate correlation with the NBSS total, as well as with the storage and voiding and the quality of life domains. The scores for the incontinence and consequences domains showed a fair to little correlation with the SF-Qualiveen results.

There was no significant correlation demonstrated between the SF-Qualiveen and EDSS scores.

## 4. Discussion

The quality of life of patients with neurogenic conditions—patients with MS in particular—is a crucial aspect of healthcare. Multiple studies have shown that having lower urinary tract symptoms has a powerful impact on the quality of life of these patients. Consequently, the use of questionnaires for patients with NLUTD is highly recommended now as a valuable addition to diagnostic procedures [3, 4].

We translated the SF-Qualiveen into Russian, culturally adapted, and validated it for the use in MS patients. The measurement properties demonstrated that the Russian version of the SF-Qualiveen is valid, reliable, and consistent. With the Russian SF-Qualiveen, Russian-speaking patients with MS can measure directly the impact of urinary symptoms on their health-related quality of life.

We made a decision to translate and validate the Short Form Qualiveen because it is more practical, easier to incorporate into research and clinical practice, and carries less patient burden. The questionnaire length has been mentioned as a limitation in the Qualiveen validation studies [10, 11].

The Russian SF-Qualiveen demonstrated a higher internal consistency for the total score (Cronbach′s alpha > 0.9) than the German (0.85) and Polish (0.86) versions validated for MS patients [13, 14]. The internal consistency of the feelings (0.93) and bother with limitations (0.79) domains was better than the frequency of limitations (0.69) and fears (0.34) domains. A similar trend was previously demonstrated by Reurs et al. for the German version (0.77 and 0.72 vs. 0.43 and 0.26, respectively) but not mentioned by Przydacz et al. for the Polish SF-Qualiveen (0.81 and 0.89 vs. 0.80 and 0.84, respectively).

The test-retest reliability showed a good reproducibility of the Russian SF-Qualiveen. The ICCs are comparable to those found in the SF-Qualiveen French, English, and German version validation studies [12, 13].

In this study, no floor or ceiling effects were recorded in the study group. Neither the highest nor the lowest possible total scores were recorded in MS patients. In the control group, 9 persons had the lowest score. Other researchers also reported no floor or ceiling effects in their patients [12–14].

The correlation between the SF-Qualiveen and NBSS scores has never been analyzed previously in MS patients. This correlation proves the external validity of the questionnaire and its correlation with clinical symptoms. The NBSS is one of the most popular assessment tools in neurourology; it has, however, only one general question about the quality of life [4]. The SF-Qualiveen provides a more detailed picture of satisfaction with life in terms of the lower urinary tract dysfunction. Used in combination, these two questionnaires complement each other. Now they are both available in Russian.

Russian is the most widely spoken Slavic language. A recent study showed that Russia is a high-risk area for MS. The latest data shows that in European Russia, the incidence of multiple sclerosis varies from 30 to 80 cases, and in Siberia, from 20 to 70 cases per 100,000 inhabitants. There are approximately 150,000 patients with MS in the country in total [25]. Moreover, around 30 million people of Russian origin live outside Russia; many of them still consider Russian as their first language. This makes the Russian diaspora one of the largest in the world and one of the most widely dispersed.

Our study does have its limitations. For instance, we were unable to measure the responsiveness of the Qualiveen and the SF-Qualiveen. This property, however, has already been established in other studies [9]. The patients we assessed here represent a highly selected cohort, treated at a single, academic center for the MS in the Ural Region of Russia; thus, our results may not be fully/directly transferrable to every clinical practice. Nonetheless, the questionnaire showed good measurement properties in the original multicenter study as well as in the validation studies [12, 14].

## 5. Conclusions

The Russian version of the SF-Qualiveen is a valid reliable tool for the evaluation of the quality of life associated with lower urinary tract symptoms in multiple sclerosis patients with a neurogenic bladder. This makes it possible to use the SF-Qualiveen in future research and clinical practice in Russia.

## Figures and Tables

**Table 1:** Summary of SF-Qualiveen results in MS patients with a neurogenic bladder.

	Minimum and maximum values	Mean (*m* ± SD)	Retest mean (*m* ± SD)	Mean difference (*m* ± SD)	ICC
Total score	0.75-3.88	2.51 ± 0.79	2.53 ± 1.01	0.02 ± 0.29	0.89
Bother with limitations domain	0.5-4.0	2.41 ± 1.01	2.39 ± 1.06	−0.02 ± 0.53	0.85
Fears domain	0.0-4.0	2.25 ± 0.88	2.25 ± 1.02	0.00 ± 0.18	0.88
Feelings domain	1.0-4.0	2.86 ± 0.86	2.85 ± 1.05	−0.01 ± 0.25	0.81
Frequency of limitations domain	1.0-4.0	2.50 ± 0.77	2.58 ± 1.13	0.08 ± 0.68	0.87

**Table 2:** SF-Qualiveen Russian version internal consistency.

	Cronbach's alpha (primary test)	Cronbach's alpha (retest)
Total score	0.91	0.89
Bother with limitations domain	0.79	0.81
Fears domain	0.34	0.37
Feelings domain	0.93	0.88
Frequency of limitations domain	0.69	0.75

**Table 3:** SF-Qualiveen Russian version external validity (Spearman correlation between SF-Qualiveen and NBSS scores).

	NBSS, total score	NBSS, incontinence domain	NBSS, storage and voiding domain	NBSS, consequences domain	NBSS, quality of life item
SF-Qualiveen, total score	0.618^∗∗^ *p* = 0.002	0.324 *p* = 0.142	0.532^∗^ *p* = 0.011	0.336 *p* = 0.126	0.838^∗∗^ *p* = 0.000
SF-Qualiveen, bother with limitations domain	0.579^∗∗^ *p* = 0.005	0.184 *p* = 0.412	0.521^∗^ *p* = 0.013	0.411 *p* = 0.058	0.783^∗∗^ *p* = 0.000
SF-Qualiveen, fears domain	0.276 *p* = 0.213	0.212 *p* = 0.344	0.110 *p* = 0.626	0.042 *p* = 0.852	0.556^∗∗^ *p* = 007
SF-Qualiveen, feelings domain	0.642^∗∗^ *p* = 0.001	0.342 *p* = 0.120	0.607^∗∗^ *p* = 0.003	0.358 0.102	0.766^∗∗^ 0.000
SF-Qualiveen, frequency of limitations domain	0.536^∗^ *p* = 0.010	0.272 *p* = 0.221	0.600^∗∗^ *p* = 0.003	0.299 *p* = 0.176	0.661^∗^ *p* = 0.01

^∗^Significant correlation at *p* < 0.05 (bilateral). ^∗∗^Significant correlation at *p* < 0.01 (bilateral).

## Data Availability

All the study data is stored in the Sverdlovsk Regional Clinical Hospital No.1 database and is available on request.
